# Impact of interprofessional education on students of the health professions: a systematic review

**DOI:** 10.3352/jeehp.2019.16.33

**Published:** 2019-10-23

**Authors:** Amy Leigh Dyess, Jordyn Shelby Brown, Natasha Dianne Brown, Katherine Merrill Flautt, Lisa Jayroe Barnes

**Affiliations:** Department of Physical Therapy, School of Health Related Professions, University of Mississippi Medical Center, Jackson, MS, USA; Hallym University, Korea

**Keywords:** Education, Attitudes of health personnel, Interprofessional, Interdisciplinary communication

## Abstract

**Purpose:**

Interprofessional education (IPE) is a concept that allows students from different health professions to learn with and from each other as they gain knowledge about their chosen professions and the professions of their colleagues. The purpose of this systematic review was to determine the effectiveness of IPE in the academic preparation of students of the health professions.

**Methods:**

A search was conducted of the PubMed and CINAHL databases using the following eligibility criteria: IPE including students from 3 or more healthcare professions, IPE exposure within academic coursework, measurement of attitudes and/or perceptions as outcomes, and quantitative reporting of results. Articles were screened by title, abstract, and full text, and data were extracted.

**Results:**

The search yielded 870 total articles. After screening, 7 articles remained for review. All studies reported a positive impact of IPE on the education of students of the health professions.

**Conclusion:**

Evidence showed that IPE activities were an effective tool for improving attitudes toward interdisciplinary teamwork, communication, shared problem-solving, and knowledge and skills in preparation for collaboration within interdisciplinary teams.

## Introduction

### Rationale

The World Health Organization has cited interprofessional education (IPE) activities as a powerful learning tool in efforts to improve healthcare delivery [1]. Effective interprofessional collaborations have resulted in decreased medical errors, increased patient satisfaction, and improved patient care [2,3]. Data collected from IPE studies may be beneficial for improving academic health professional programs, preparing students for their chosen profession outside of the classroom, and refining patient-centered care [3].

Due to the complex nature of the health issues that many patients face, there is an increasing need for interprofessional collaboration among healthcare practitioners. Interprofessional practice contributes to knowledge of effective communication, the importance of distinguishing team roles, and efficient solutions for resolving conflict [4]. Healthcare faculty members across the United States have recognized the importance and benefits of implementing IPE in the academic curriculum [1].

IPE is a concept that allows students from different health professions to learn with and from each other as they gain knowledge about their chosen professions, as well as the professions of their future colleagues [3,5]. Various IPE activities should occur in an environment that supports collaborative learning to better facilitate interprofessional practice in the clinical care of patients [3,5].

### Objectives

The objective of this systematic review was to determine the effectiveness of IPE among students of the health professions.

## Methods

### Ethics statement

This report examined previously published work. Therefore, it did not require approval from an institutional review board.

### Study design

The preparation of this systematic review followed the Preferred Reporting Items for Systematic Reviews and Meta-Analyses (PRISMA) guidelines, which outline specific criteria to be included in written format. The PRISMA tool consists of 27 items that have been created to ensure that essential information is expressed [6].

### Eligibility criteria

Articles that were included in this review required participation in IPE activities by students from 3 or more healthcare professions, and the IPE exposure must have been included within academic coursework. Additionally, the studies must have reported quantitative measures of student attitudes and/or perceptions about their IPE experiences as outcomes.

### Information sources/search

A search of the literature was performed in October 2018 using the PubMed and CINAHL databases. The search included terms related to healthcare, healthcare professionals, students, and IPE. Searches were limited to articles in the English language published within the last 10 years.

### Study selection

Duplicate articles in both databases were eliminated from one set of the search results. Titles were screened by 4 of the authors divided into 2 groups. If title screening resulted in a tie, it was presented to the opposite group for a final decision. This process was followed by a screening of the abstracts utilizing the same methodology. After the abstracts were screened, the full text of the remaining articles was screened by all authors in order to determine which articles would be included in the review.

### Data collection process and data items

The articles chosen from the screening process were analyzed by the authors for data extraction, and the findings were discussed with the group. The data extracted from the articles included the number of participants and the specific type of health profession training program in which they were enrolled, the type of interprofessional learning activity that was included, the specific outcome measures used, and the authors’ findings.

### Risk of bias assessment

The quality assessment tool for before-after (pre-post) studies with no control group was used to assess the quality of the articles included [7]. This is a 12-question tool developed to allow reviewers to systematically assess the internal validity of a study. Each question was answered with yes, no, or not reported, and the total number of affirmative responses was recorded.

## Results

### Study selection

**Fig. 1.**
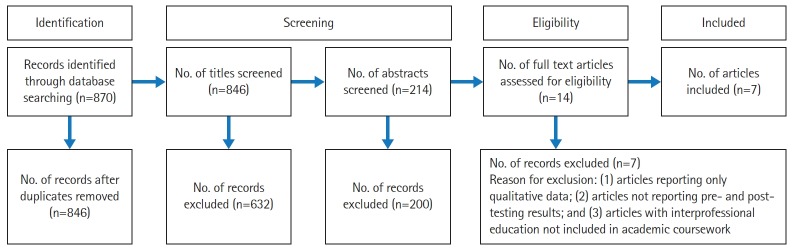
Preferred Reporting Items for Systematic Reviews and Meta-Analyses diagram.

The article search yielded 870 articles, of which 24 were eliminated due to duplication. The titles of the resultant 846 articles were screened; through this process, 632 articles were eliminated, resulting in 214 articles for the abstract screening. Screening of the individual abstracts yielded 14 articles that underwent full-text screening. Following the complete screening process, 7 articles were selected to be included in the review (Fig. 1).

### Study characteristics

The 7 studies consisted of participants from a wide variety of health professions who took part in IPE activities within their academic coursework. The number of health professions included within each study ranged from 3 to 11, and the number of participants ranged from 30 to 291 (Table 1).

### Risk of bias

The results from the quality assessment tool for before-after (pre-post) studies with no control group are shown in Table 2. The quality scores ranged from 7 to 10 out of 12, with a mean of 8.7.

## Discussion

### Summary of evidence

The evidence from this review showed that IPE activities were an effective tool for improving attitudes toward interdisciplinary teamwork, communication, shared problem-solving, and knowledge and skills in preparation for collaboration with other members of interdisciplinary healthcare teams. All 7 studies yielded significant results in regard to IPE among students of the health professions and its positive impact on students’ attitudes related to interprofessional teamwork [3,5,8-12]. Positive results were found among several healthcare professions, supporting the incorporation of IPE in the academic preparation of future healthcare providers across disciplines. Although varied outcome measures were utilized across the studies, each showed some impact on attitudes and self-perceptions of interprofessional teamwork in the delivery of healthcare services.

The works by Ruebling et al. [11] in 2014 and Leithead et al. [8] in 2018 each incorporated outcome measures that included items addressing interpersonal communication and problem-solving, which demonstrated significant changes after IPE activities. Effective communication and collaborative efforts to find solutions to clinical issues across healthcare professions is essential for achieving the best outcomes for patients and establishing a positive work environment. These findings are supported by the works by Paige et al. [9] in 2014 and Cino et al. [5] in 2018, who also found significant improvements in constructs related to communication among the healthcare team, while Paige et al. [9] in 2014 and Stubbs et al. [12] in 2017 suggested that IPE may be beneficial for improvements in shared problem-solving. In addition to benefits in teamwork, communication, and shared problem-solving, the studies by Renschler et al. [3] in 2016 and Cino et al. [5] in 2018 each suggested that IPE activities may improve knowledge and skills among students of the health professions, which may ultimately benefit the consumers of healthcare services.

### Limitation

Publications within this review were limited to the English language; therefore, important work published in other languages may not have been represented. Additionally, studies that consisted of participants from only 2 healthcare professions were excluded, which may also have led to the omission of important works.

### Conclusion

The information gleaned from these studies supports the incorporation of IPE in the educational preparation of healthcare professionals. Important aspects of healthcare delivery, such as working within a team environment, the ability to work together in a way that promotes shared decision-making to find positive solutions, effective interpersonal communication, and excellent knowledge and skills, may be positively influenced by the utilization of IPE activities across health professions within academic programs.

## Figures and Tables

**Table 1.** Data summary

References	Student type (no. of participants)	Intervention	Outcome measures	Results
Cino et al. [5] (2018)	Dental hygiene (10); nursing (37); medical laboratory technician (28)	Students participated in a questionnaire determining self-efficacy for IPE before and after participation in an interprofessional ethics activity.	Self-efficacy for interprofessional learning	Six questions yielded significant improvements in self-efficacy for IPE learning
				Teamwork (P=0.023)
				Multiple professions roles (P=0.031)
				Patient benefits of team care (P=0.043)
				Objectives of interprofessional learning (P=0.019)
				Quality of interprofessional team work (P=0.008)
				Interprofessional learning goal achievement (P=0.012)
Leithead et al. [8] (2018)	Medical (70 IPT/43 RIPLS); undergraduate nursing (40 IPT/27 RIPLS); nurse anesthesia (42 IPT/29 RIPLS)	Students participated in IPE involving a high-fidelity simulation of the operating room over the course of 3 years.	IPT	Significant improvement overall was reported on IPT following the intervention (P<0.001)
			RIPLS	Significant improvement overall was reported on RIPLS following the intervention (P<0.001)
Paige et al. [9] (2014)	Nursing (18); certified registered nurse anesthetist (20); medical (28)	Students participated in IPE involving a high-fidelity simulation of the operating room.	Likert-type items measuring self-efficacy and team performance	Eleven out of the 15 items showed significant improvement following the intervention including items related to individual task responsibilities, communication, and teamwork (P<0.001)
Pinto et al. [10] (2018]	Medical (70); occupational therapy (34); physical therapy (28); physician assistant (12); nursing (44)	Students completed a stroke simulation activity along with pre- and post-test questionnaires regarding the IPE.	IPE collaborative competency self-assessment tool	Following the intervention, overall results indicated significant improvement in interprofessional values and interprofessional interactions
				Values domain (P<0.0001)
				Interactions domain (P=0.0003)
Renschler et al. [3] (2016)	Osteopathic medicine (1st 19/2nd 21); nursing (1st 30/2nd 41); health science (1st 7/2nd 6); speech-language (1st 18/2nd 17); athletic training (1st 4/2nd 0); exercise sciences (1st 3/2nd 0)	Student participation in an interprofessional geriatric home visit program for a 1-sem versus a 2-sem IPE activity.	Attitudes Towards Health Care Teams Scale	1st sem: led to significant improvements following the intervention (P=0.00)
				2nd sem: showed no significant improvements
			Team Skills Scale	1st sem: showed significant improvement in team skills (P=0.00)
				2nd sem: reported significant improvements (P=0.01)
			-	Between-group comparisons showed more significant improvements in the 1-sem program than in the 2-sem program (P<0.05)
Ruebling et al. [11] (2014)	Athletic training (15); clinical laboratory (9); cytotechnology (2); health information management (2); investigative medical sciences (15); nuclear medicine (4); nursing (115); nutrition and dietetics (18); occupational therapy (20); physical therapy (83); Radiation therapy (8)	Questionnaire completed by students who participated in a sem-long IPE course in interdisciplinary teams.	RIPLS	Significant improvements after the introductory IPE course (P=0.05)
				Significant improvements after the IPE course (P<0.001)
			University of West England Interprofessional Questionnaire	Improvements were noted after the IPE course (P=0.01)
				Significant improvements after the IPE course (P<0.001)
Stubbs et al. [12] (2017)	Dentistry; dietetics; divinity; medicine; nursing; occupational therapy; pharmacy; public health; social work; speech and hearing science (30)	Students completed a questionnaire before the IPE program, after IPE training and upon completion of the IPE consisting of didactic and community service work.	ISVS	The ISVS contained 3 sub-scales: SPA, CWO, VWO
				SPA: After completion significant improvements were reported (P=0.005)
				CWO: After completion significant improvements were reported (P<0.0001)
				VWO: After completion significant improvements were reported (P=0.001)

IPE, interprofessional education; IPT, Interprofessional Teamwork Scale; RIPLS, Readiness for Interprofessional Learning Scale; Sem, semester; ISVS, Interprofessional Socialization & Valuing Scale. SPA, self-perceived ability to work with others; CWO, comfort working with others; VWO, value of working with others.

**Table 2.** Risk of bias

Quality assessment tool	1	2	3	4	5	6	7	8	9	10	11	12	Total
Cino et al. [5]	Yes	No	NR	NR	Yes	Yes	Yes	No	Yes	Yes	No	Yes	7
Leithead et al. [8]	Yes	Yes	Yes	No	Yes	No	Yes	No	Yes	Yes	Yes	Yes	9
Paige et al. [9]	Yes	No	Yes	No	No	Yes	Yes	No	Yes	Yes	Yes	Yes	8
Pinto et al. [10]	Yes	Yes	Yes	Yes	Yes	No	Yes	No	Yes	Yes	No	Yes	9
Renschler et al. [3]	Yes	Yes	Yes	Yes	Yes	Yes	Yes	Yes	NR	Yes	No	Yes	10
Ruebling et al. [11]	Yes	Yes	Yes	Yes	Yes	Yes	Yes	No	No	Yes	No	Yes	9
Stubbs et al. [12]	Yes	No	Yes	Yes	No	Yes	Yes	No	Yes	Yes	Yes	Yes	9

Criteria: 1: study question; 2: eligibility criteria and study population; 3: study participants representative of clinical populations of interest; 4: all eligible participants enrolled; 5: sample size; 6: intervention clearly described; 7: outcome measures clearly described, valid, and reliable; 8: blinding of outcome assessors; 9: follow-up rate; 10: statistical analysis; 11: multiple outcome measures; 12: group-level interventions and individual-level outcome efforts. NR, not reported.

## Supplementary materials

Data files are available from https://doi.org/10.7910/DVN/K3BKTG

Supplement 1. Audio recording of the abstract.
